# A Comparison of the Antibacterial and Antifungal Activities of Thiosulfinate Analogues of Allicin

**DOI:** 10.1038/s41598-018-25154-9

**Published:** 2018-04-30

**Authors:** Roman Leontiev, Nils Hohaus, Claus Jacob, Martin C. H. Gruhlke, Alan J. Slusarenko

**Affiliations:** 1 Department of Plant Physiology, RWTH Aachen University, 52056 Aachen, Germany; 2 Division of Bioorganic Chemistry, School of Pharmacy, Saarland University, 66041 Saarbrücken, Germany

## Abstract

Allicin (diallylthiosulfinate) is a defence molecule from garlic (*Allium sativum* L.) with broad antimicrobial activities in the low µM range against Gram-positive and -negative bacteria, including antibiotic resistant strains, and fungi. Allicin reacts with thiol groups and can inactivate essential enzymes. However, allicin is unstable at room temperature and antimicrobial activity is lost within minutes upon heating to >80 °C. Allicin’s antimicrobial activity is due to the thiosulfinate group, so we synthesized a series of allicin analogues and tested their antimicrobial properties and thermal stability. Dimethyl-, diethyl-, diallyl-, dipropyl- and dibenzyl-thiosulfinates were synthesized and tested *in vitro* against bacteria and the model fungus *Saccharomyces cerevisiae*, human and plant cells in culture and *Arabidopsis* root growth. The more volatile compounds showed significant antimicrobial properties via the gas phase. A chemogenetic screen with selected yeast mutants showed that the mode of action of the analogues was similar to that of allicin and that the glutathione pool and glutathione metabolism were of central importance for resistance against them. Thiosulfinates differed in their effectivity against specific organisms and some were thermally more stable than allicin. These analogues could be suitable for applications in medicine and agriculture either singly or in combination with other antimicrobials.

## Introduction

**Figure 1:**
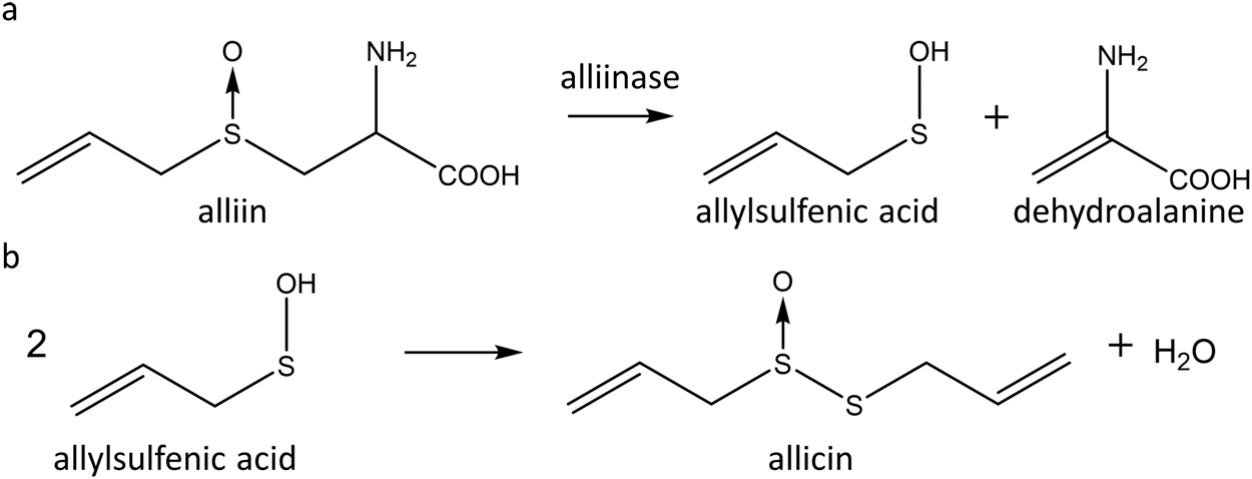
Biosynthesis of allicin from S-allyl cysteine sulfoxide (alliin). The enzyme alliinase (a C-S lyase, E.C. 4.4.1.4.) catalyzes the formation of allylsulfenic acid and dehydroalanine (**a**), whereupon two molecules of allylsulfenic acid condense spontaneously to yield one molecule of allicin (**b**).

Garlic has been used since ancient times for its health beneficial properties and modern research has provided a scientific basis for this practice^1–3^. Garlic compounds have been shown to decrease cholesterol and fatty acid levels in the blood^4–6^ and lower blood pressure^7–11^; thus, garlic consumption can contribute to the prevention of cardiovascular diseases^12^. Anti-tumour activities of garlic compounds have been demonstrated, providing for a potential use in cancer-therapy and prevention^13,14^. Another very important garlic property is the antimicrobial activity observed in raw garlic extract. The main anti-bacterial compound of fresh garlic is allicin, a thiosulfinate with two allyl groups as carbon chains (diallylthiosulfinate)^15,16^. Besides bacteria, the effects of allicin have been investigated against fungi, protozoa and viruses^17–19^. Methicillin-resistant *Staphylococcus aureus* (MRSA) isolates were also shown to be susceptible to allicin^20^. Allicin is produced from the non-protein amino acid alliin (S-allylcysteine sulfoxide) upon tissue damage in a reaction that is catalyzed by the enzyme alliinase (Fig. 1). Structurally analogous thiosulfinates are produced in nature by other *Allium* and *Petiveria* spp.^21^, and antimicrobial activity has been reported for this group of compounds^22–24^. Unlike conventional antibiotics, allicin is volatile and can kill bacteria via the gas phase^17^. This is particularly interesting since many lung-pathogenic bacteria are susceptible to allicin^25,26^. Although allicin is also toxic to human cells^27,28^, the successful treatment of tuberculosis by breathing in the vapour from crushed garlic preparations was reported in the pre-antibiotic era^29,30^.

Thiosulfinates are disulfide-S-monoxides and as products of the condensation of two sulfenic acids, can be viewed as ‘sulfenic acid anhydrides’^31^. In the laboratory, allicin can be synthesized most effectively by oxidation of diallyldisulfide (DADS) with H_2_O_2_ in the presence of an organic acid catalyst that is first oxidized to the corresponding peroxy-acid, e.g. performic acid or peracetic acid^23,32^.

**Figure 2:**
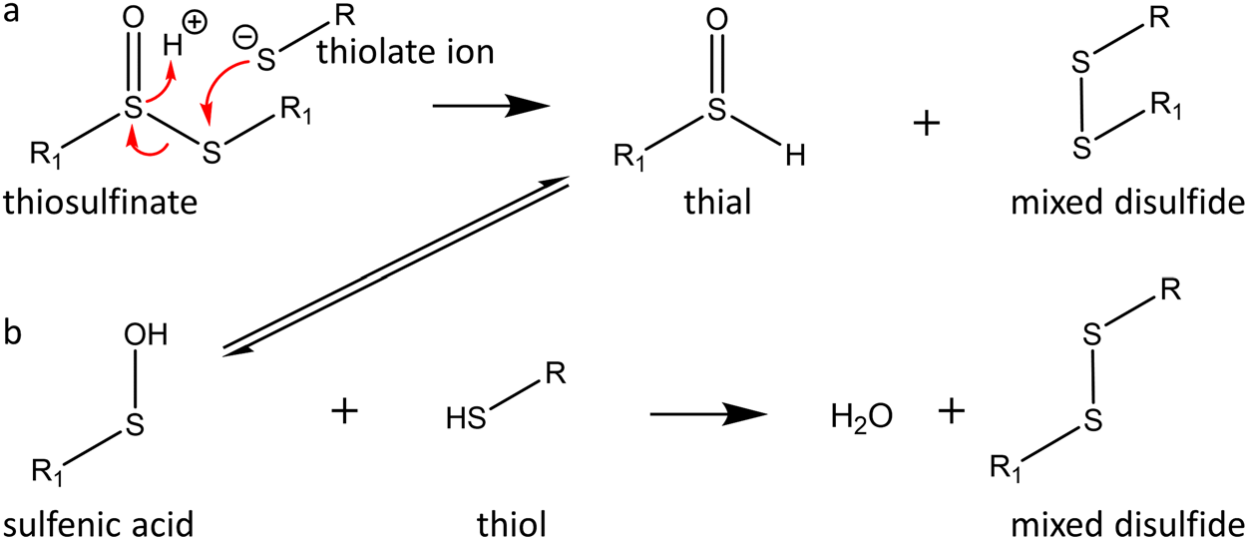
Reaction mechanism of a thiol with a thiosulfinate. The reaction leads directly (**a**), and indirectly (**b**), to the formation of a mixed disulfide, which under some conditions may react further with RSH in a thiol disulfide exchange reaction to form RSSR and R_1_SH.

The reactivity of thiosulfinates towards thiol-groups is an important component of their antimicrobial activity^15,23,33,34^. The electron-withdrawing effect of the *O*-atom creates an electrophilic sulfur centre which reacts readily with thiols, or more specifically, with thiolate ions (Fig. 2), thereby forming an *S*-allylmercapto adduct. Thus, many enzymes with catalytically important thiol-groups are oxidized and inhibited when exposed to allicin^33^, whilst, a few enzymes are activated upon oxidation by allicin, for instance fructose-1,6-bisphosphatase from chicken liver^35^.

To understand allicin’s cellular mode of action in more detail, a proteome-wide investigation in *Escherichia coli* was performed to identify the proteins oxidized by allicin exposure. After cells were treated with 0.79 mM allicin, 73 *S*-thioallylated proteins were identified, including some essential enzymes of primary metabolism^36^. Furthermore, allicin reacts with low-molecular weight cellular thiols such as glutathione (GSH), shifting the GSH-based cellular redox-potential to a more oxidized state. Indeed, it was recently shown for *Saccharomyces cerevisiae* that treatment with allicin altered the ratio of reduced (GSH) and oxidized glutathione (GSSG) into a range that would predict induction of apoptosis and this was confirmed by cytological and genetic methods^37^. In yeast, GSH synthesis is regulated by the Yap1p transcription factor, which has oxidation-sensitive cysteines and coordinates the oxidative stress response by regulating the expression of response genes. For example, Yap1p regulates the expression of the GSH biosynthetic genes *GSH1*, *GSH2* and glutathione reductase (*GLR1*), which utilizes NADPH to reduce GSSG to GSH. Allicin was shown to oxidize critical cysteines in Yap1p and *Δyap1* and *Δglr1* mutants were shown to be hypersensitive to allicin^38^. This situation is analogous to the essential role of GSH in resistance of yeast to dipyridyl disulfide, which is also a highly specific reagent for thiol groups, showing pronounced antifungal activity^39^.

Besides being redox active, allicin is also quite lipophilic. The calculated log*P* value of allicin is 1.35, indicating that allicin is membrane permeable and its antimicrobial activity is certainly facilitated by its ready entry into cells^40^. At the same time, it has been shown that allicin is able to form transient pores in artificial and in bio-membranes, which perhaps accounts for its reported synergy with membrane-active antibiotics such as amphotericin B and polymixin B^41^.

Overall, this magnitude of data shows that allicin is on the way to become a well characterized natural product with potential to be used both in medicine and agriculture. Allicin is unstable in storage and degrades rapidly at temperatures above 80 °C^17,42^. However, allicin and its derivatives have been discussed as lead compounds for new antibiotics^24,43^, but very little is known about the biological activities of other thiosulfinates. Nevertheless, there is some promising albeit limited data on some allicin-derivatives which have been tested as inhibitors for cysteine proteases in the parasitic protozoa *Plasmodium falciparum* and *Trypanosoma brucei*^44^. Furthermore, along with allylisothiocyanate (AITC), DMTS (frequently referred to as methyl methanethiosulfinate, MMTSO) was described as one of the most important antimicrobial compound in cabbage plants for defence against microbial pathogens^45,46^. In garlic therefore, thiosulfinates other than allicin, although quantitatively more minor, may still be of considerable biological activity and interest.

In the work reported here we have synthesized a series of simple thiosulfinates, based on allicin as the lead compound, and evaluated their chemical stability and antimicrobial effectivity. Here, dimethyl- (DMTS), diethyl- (DETS), diallyl- (DATS, allicin), dipropyl- (DPTS) and dibenzyl- (DBTS) thiosulfinates form a series of increasing molecular mass and hydrophobicity which would be expected to affect physical characteristics such as rate of diffusion, volatility and membrane accessibility/permeability, all of which may be expected to have an influence on the biological properties of the molecules. We showed that some of the thiosulfinates are active as a vapour and that as little as one hour exposure to allicin vapour was inhibitory to microbial growth. We investigated a series of yeast mutants affected in GSH metabolism and protein disulfide reduction and showed similar responses to those for allicin, suggesting a similar mode of action. We confirm that the thiosulfinate moiety is important for antimicrobial activity but that this activity is modified by the surrounding substituent groups. The compounds tested are all quite ‘allicin-like’ in structure and mostly naturally occurring, and the results are discussed in terms of the merits and the potential of such thiosulfinates for applications in medicine and agriculture.

## Results and Discussion

### Comparison of the physical properties of DMTS, DETS, DATS, DPTS and DBTS

**Figure 3:**
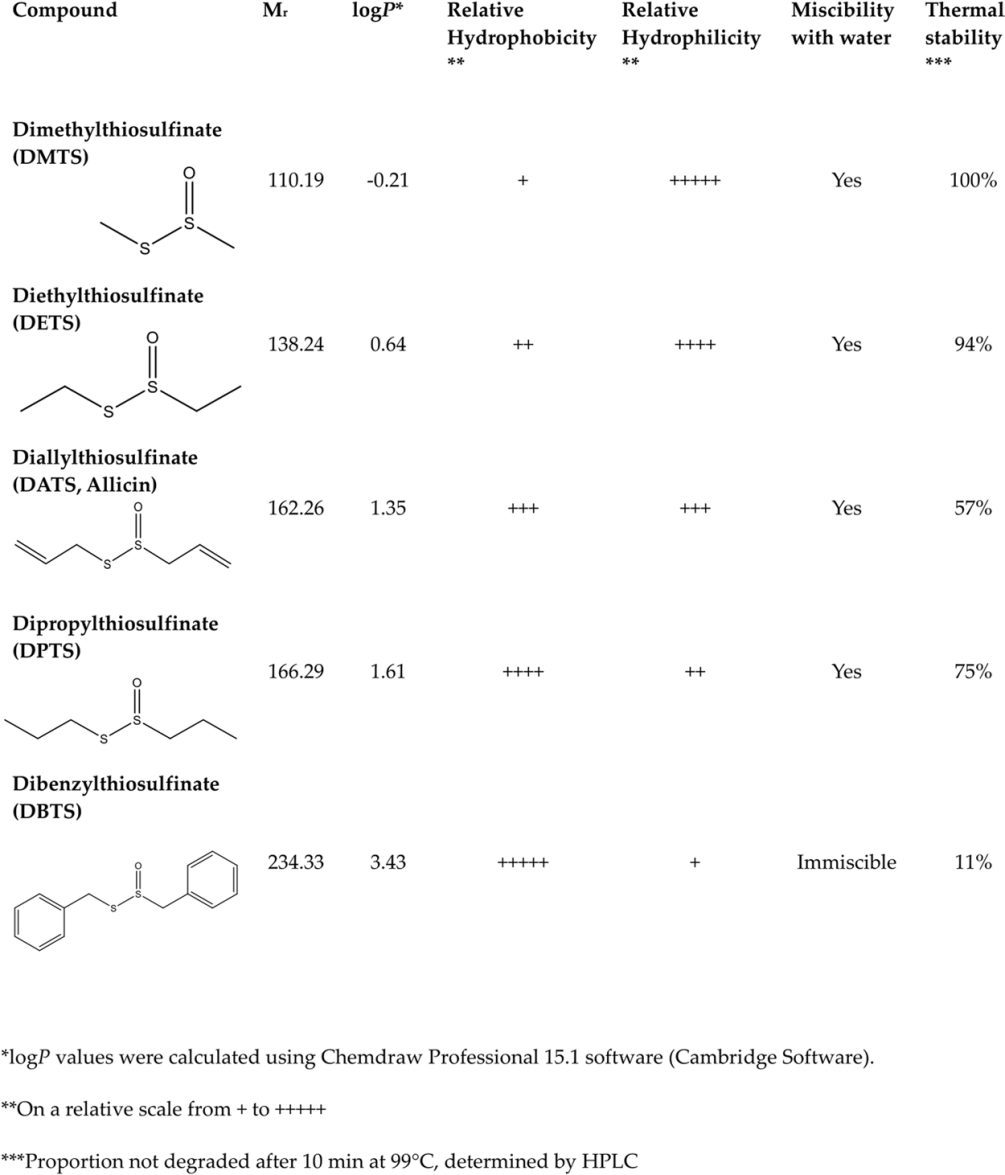
Physical properties of different thiosulfinates.

The structures and the physical properties of the test substances dimethyl- (DMTS), diethyl- (DETS), diallyl- (DATS, allicin), dipropyl- (DPTS) and dibenzyl- (DBTS) thiosulfinate are summarized in Fig. 3. As an indication of the relative membrane permeabilities the calculated log*P* values, also known as log K_ow_ for the log of the partition coefficient between octanol and water, were calculated using the software Chemdraw Professional 15.1. DMTS was the most hydrophilic compound tested (log *P* = −0.21) while DBTS was the most hydrophobic (log *P* = 3.43). DBTS was immiscible with water, methanol and ethanol and decomposed in DMSO, but was found to be soluble and stable in dimethyl formamide (DMF), in which it was dissolved for use in experiments (see Materials and Methods). DMTS, DETS, DATS and DPTS were sufficiently water soluble to be used in aqueous solutions.

### Comparison of MICs, MBCs and MFCs for DMTS, DETS, DATS and DPTS

We set out to survey a range of microorganisms with respect to the effects of the thiosulfinates and based on our previous experience with allicin we chose *E. coli* as an example of an enteric bacterium, *P. fluorescens* and *P. syringae 4612* as examples of relatively allicin-resistant and allicin-susceptible strains, respectively, *M. luteus* as a Gram-positive example and the Euroscarf yeast reference strain BY 4742 as a model fungus. International standard EUCAST test procedures were used to determine the minimal inhibitory concentration (MIC) and minimal bactericidal or fungicidal concentration (MBC, MFC), respectively, for the different thiosulfinates with selected bacteria and *Saccharomyces cerevisiae*. It should be noted that the use of µg mL^−1^, rather than equimolar concentrations, is an unfortunate anachronism of the internationally used standard test protocol^47^. MICs for the bacteria tested ranged from 8–256 µg mL^−1^ and MBCs from 16–256 µg mL^−1^ for the various thiosulfinates (Table 1).

**Table 1:** MIC and MBC values (µg mL^−1^) for the different thiosulfinates in the bacteria tested and MIC and MFC for yeast.

		Gram-positive			Gram-positive	Yeast
		*Escherichia coli* K12 *Ec*	*Pseudomonas fluorescens* *Pf*−01	*Pseudomonas syringae* pv. *phaseolicola* 4612 *Ps*4612	*Micrococcus luteus* *Ml*	*Saccharo-myces* *cerevisiae* BY4742 *Sc*
Thio-sulfinate	Conc. (µg mL^−1^)					
DMTS	MIC MBC	64 64	16 32	16 16	64 64	16 MFC 16
DETS	MIC MBC	64 64	128 256	8 16	32 32	8 MFC 8
DATS	MIC MBC	32 32	128 256	16 16	16 32	2 MFC 4
DPTS	MIC MBC	32 32	256 256	32 64	32 32	2 MFC 4

The values are given as the highest value out of three replicates.

As is apparent from Table 1, the model fungus *S. cerevisiae* (*Sc*, baker’s yeast) was more susceptible to thiosulfinates when compared to the bacteria tested. At the same time, DATS (allicin) and DPTS were the most effective compounds, with a MIC of 2 µg mL^−1^ and a MFC = 4 µg mL^−1^ for both thiosulfinates.

Indeed, with only few exceptions, DATS was the thiosulfinate most effective against all test organisms; however, in some cases other thiosulfinates showed equal or marginally better activity in the EUCAST test procedure. Nonetheless, our data does not reveal any universal trends of antibiotic effectivity, such as a distinctive structure-activity relationship or a strong correlation between log*P* and activity, as may have been anticipated. Thus, for *E. coli* there seemed to be a slight increase in effectivity from 64 to 32 µg mL^−1^ (MIC = MBC) as one progresses along the M_r_ series DMTS, DETS, DATS and DPTS whereas for *Pseudomonas fluorescens* the reverse trend was apparent with a decreasing effectivity from DMTS, DETS, DATS to DPTS (Table 1). Interestingly, DMTS was more active against *P. fluorescens* than DATS, with a MIC of 16 µg mL^−1^ and a MBC of 32 µg mL^−1^-compared to DATS with a MIC of 128 µg mL^−1^ and a MBC = 256 µg mL^−1^. Notably, most of the thiosulfinates were active in the low micromolar to low millimolar range, e.g. the MIC for allicin and yeast was 2 µg mL^−1^ (=12 µM) and for *P. fluorescens* was 128 µg mL^−1^ (=0.8 mM).

### Comparison of antibacterial activities of thiosulfinates using a Petri-plate-diffusion test

**Figure 4:**
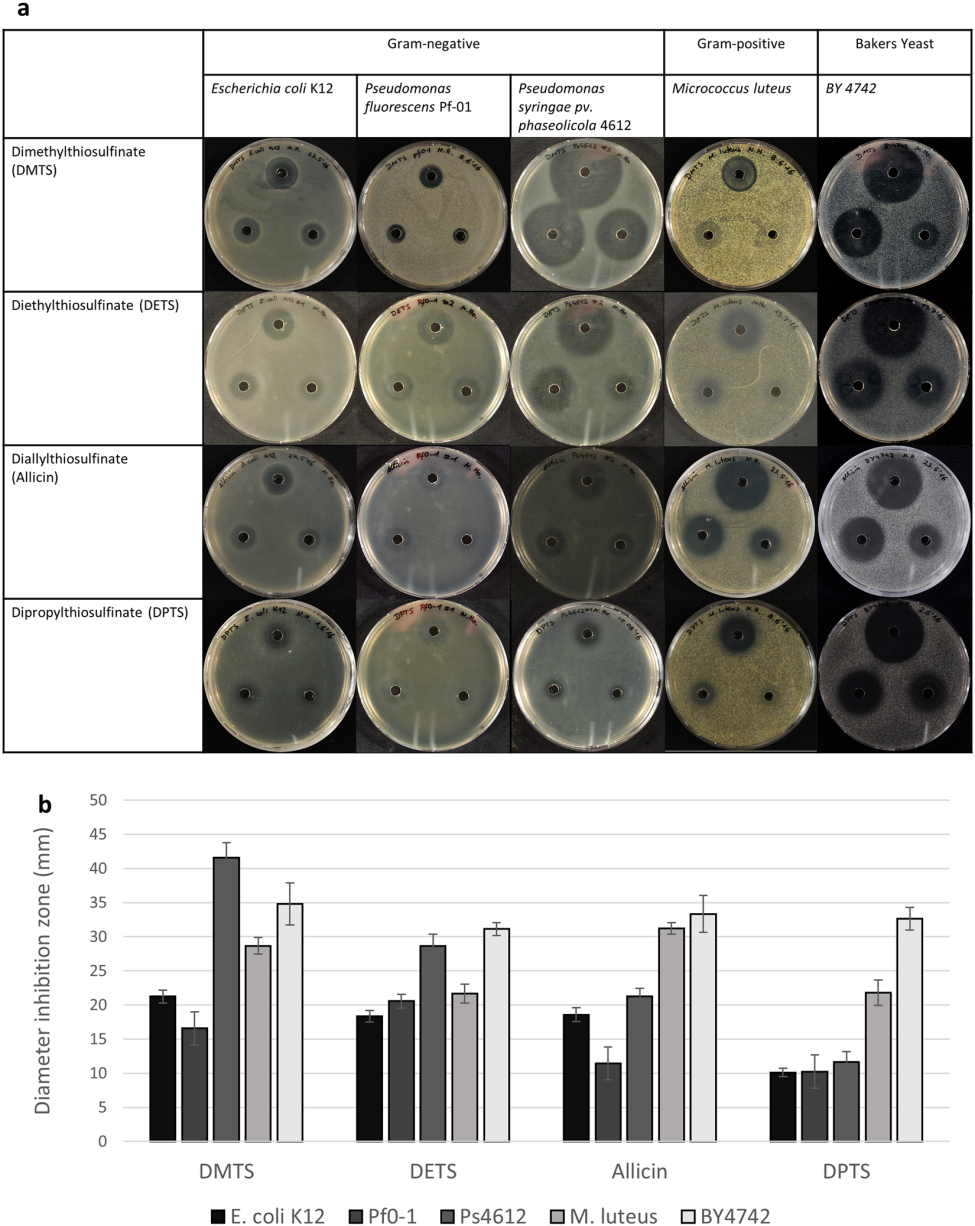
Plate inhibition zone assay showing the antimicrobial activity of different thiosulfinates against various bacteria and yeast. (**a**) Representative pictures for Gram-negative bacteria (*E. coli* K12, *Pseudomonas* spp.), Gram-positive bacteria (*M. luteus*) and yeast BY4742 cells. Cells were incorporated in 50 °C warm agar and poured into a Petri-dish. The upper well contained 20 µL of 8 mM solution, the hole at the left side of each Petri-dish contained 20 µL of 4 mM solution and the hole at the right side of the Petri-dish contained 20 µL of 2 mM solution of each compound tested. (**b**) The diameter of inhibition zone was measured for the hole containing 20 µL of 8 mM solution of the test compound. Error bars show standard deviation about the mean, n = 9.

In this part of the investigation, bacteria-seeded agar was used. A bacterial suspension was mixed in the agar medium just above gelling temperature and then poured rapidly into the Petri-plate to give an even distribution of cells throughout. Holes were cut with a cork borer and 20 µL of test solution were pipetted into each well. The usefulness of this test for assessing antimicrobial activity of novel test substances is described in^48^. A crucial prerequisite for this assay is adequate water-solubility of the compound, so it is able to diffuse through the water-based agar-solidified medium. Since DBTS is not water-soluble and needs to be dissolved in DMF, we could not perform the plate-inhibition zone assay with it. DMTS, DETS, DATS and DPTS showed antibiotic activity against all bacteria in this study, resulting in clear inhibition zones, the size of which was dose-dependent (Fig. 4A,B).

Each test substance will diffuse into the agar from the central well and establish a concentration gradient over the time of the experiment. Thus, each substance can be compared for relative efficacy against different test organisms, but the efficacy of the substances cannot be compared with each other because of their assumedly different diffusion rates. A further cautionary note is, that since *E. coli* cells are cultivated at 37 °C and all other organisms at 28 °C, the diffusion behaviour of the test substances for *E. coli* will also not be comparable to that in tests with the other organisms. Still, within the constraints outlined above, it can be seen that, for each given test substance, relative antibiotic effectivity differed between the different organisms exposed to them. Therefore, considering antibacterial activity first, DMTS was most effective in this test system against *P. syringae 4612* and least effective against *P. fluorescens* whereas DATS was most effective against *Micrococcus luteus* and least effective against *P. fluorescens* (Fig. 4A,B). All the thiosulfinates proved very effective against yeast BY4742 cells and resulted in relatively large inhibition zones in comparison to those for bacteria. This is in agreement with the results of the EUCAST procedure, which also showed yeast to be more sensitive to thiosulfinates when compared to the bacteria tested (Table 1). In control plates without thiosulfinate, bacteria grew up to the edge of the well as a continuous lawn.

### Antimicrobial effects of thiosulfinates via the gas phase

**Figure 5:**
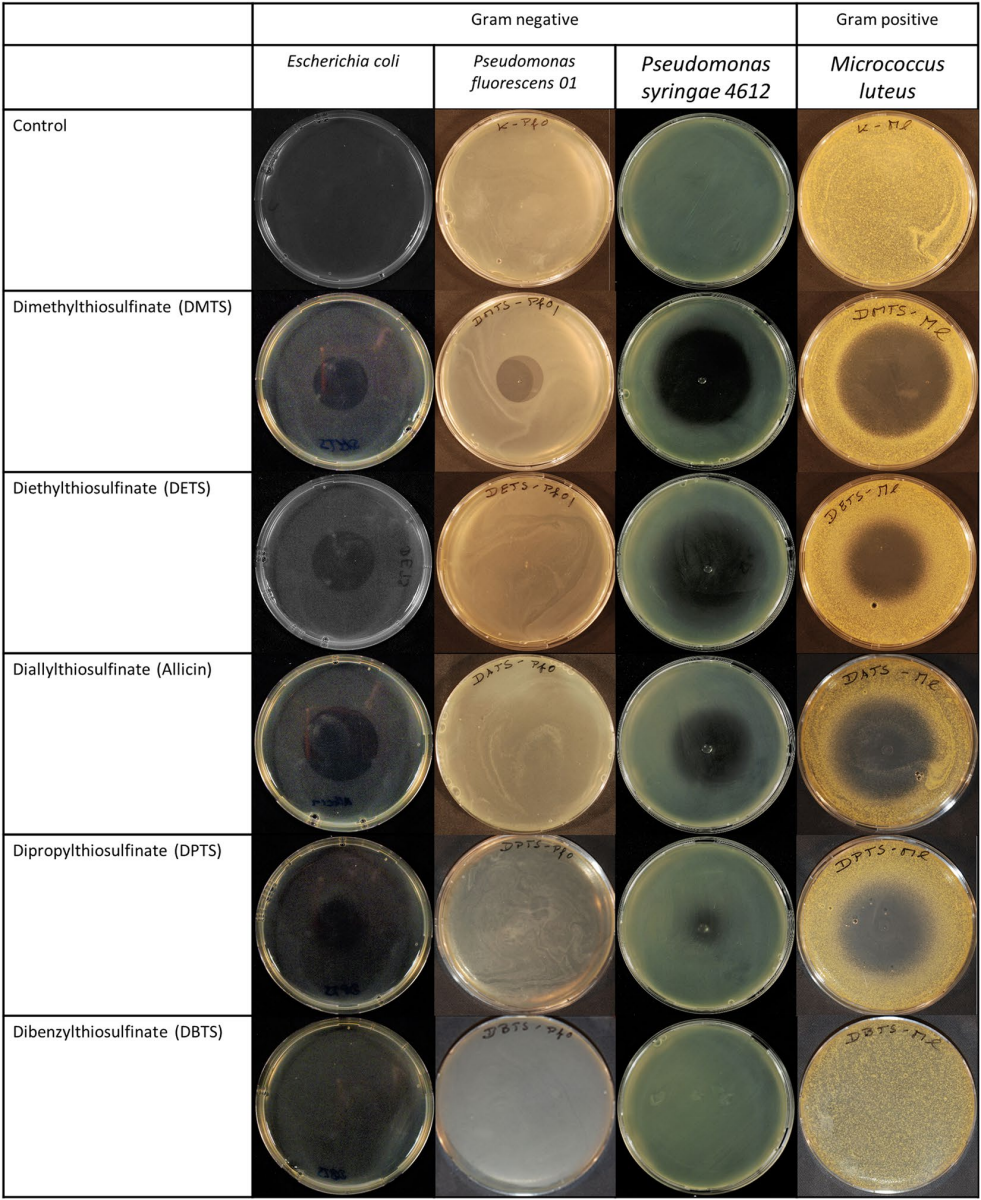
DMTS, DETS, DATS, DPTS, but not DBTS, show antibiotic activity via the gas phase. A 20 µL drop of an 80 mM solution of the test thiosulfinate was placed in the lid of a Petri-dish and the base with bacteria-seeded agar inverted over it. Thus, the agar did not come into contact with the droplet. Inhibition of growth was visible as a halo with reduced bacterial growth.

To investigate the antibiotic activity of DMTS, DETS, DATS, DPTS and DBTS via the gas phase, a 20 µL drop of 80 mM test solution was placed in the centre of a Petri-dish lid and the Petri-dish base, containing medium seeded with bacteria, was inverted above the lid as previously described^17^. Thus, there was no contact between the test solution and the agar itself except by diffusion through the air. With the exception of DBTS, which was presumably not sufficiently volatile to achieve inhibitory concentrations, all thiosulfinates produced an inhibition zone above the droplet in the Petri plate lid for *E. coli*, *P. syringae 4612* and *M. luteus*. Interestingly, only DMTS, which is presumably the most volatile of the thiosulfinates under investigation, was able to inhibit *P. fluorescens* via the gas phase (Fig. 5).

It is perhaps surprising in this experiment to see such clear zones of inhibition with fairly sharp borders. We interpret this as reflecting the concentration gradient of the thiosulfinates diffusing away from the central drop into the still air above it in the closed Petri dish, and a tight threshold inhibition concentration.

**Figure 6:**
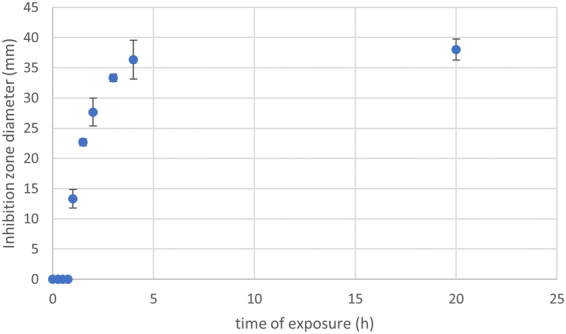
Allicin (20 µL droplet of 80 mM solution) shows inhibitory effects against *E. coli* in this test dependent on exposure time, with maximum inhibition reached after only four hours exposure.

To test how much time was required for allicin to diffuse through the gas phase and achieve an inhibitory concentration at the seeded agar, a time-resolved experiment with allicin and *E. coli* was carried out. After a given exposure time the seeded agar plate was placed over a new lid without allicin solution and incubated overnight. The results show that as little as one hour of exposure to allicin already leads to an effective growth inhibition of bacteria above the drop. An exposure of only four hours was sufficient to achieve maximum inhibition, since exposure for 20 hours did not result in a bigger inhibition zone (Fig. 6).The complete lack of an inhibition zone for the 15, 30 and 45 minute time points presumably reflects the ‘deadtime’ required for the allicin wave to diffuse across the airspace between the drop and the agar surface and reach the minimum inhibitory concentration. Just 15 minutes later at the 1 h time point an inhibition zone approximately one third of the maximum diameter was achieved.

### Comparison of antibacterial activities of thiosulfinates in drop tests

In this procedure, the test substance was incorporated at a given concentration into medium kept just above gelling temperature and the plates were poured immediately. Aliquots (10 µL) of a 10^n^ dilution series of a log-phase culture of the test bacteria were pipetted as discrete spots onto the medium. Inhibition can be seen in comparison to growth on control plates without test substance. *E. coli* cells (Gram-negative) were hardly inhibited by 100 µM DMTS, DPTS or DBTS whereas allicin and DETS caused a high degree of inhibition after 36 h. In the agar diffusion test, *E. coli* cells were inhibited by all test substances and this result demonstrates the importance of not relying on the conditions of a single test when assessing the antimicrobial effectivity of test compounds. Thus, the standard EUCAST procedure uses a low titre of cells in stationary culture, the agar diffusion test works with a concentration gradient, the drop test incorporates the substance at fixed concentrations and different cell densities are tested, whereas shake culture exposes the test cells under conditions of continuous agitation and high aeration. Furthermore, divergent results for the different test scenarios, illustrate that only the relevant test situation in the real world (clinical or agricultural situation) will be definitive.

**Figure 7:**
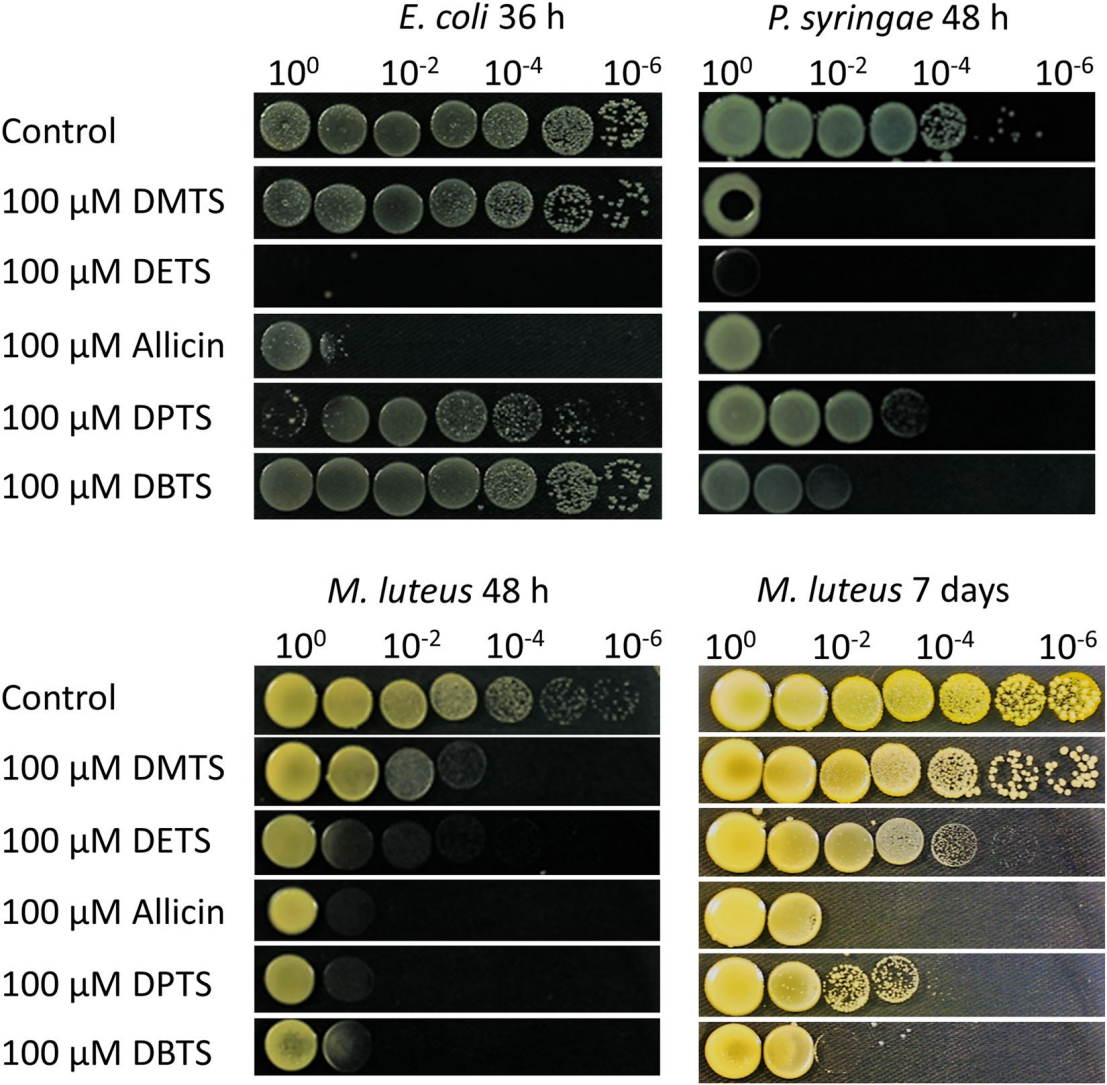
Drop test illustrating the relative inhibitory activities of the thiosulfinates incorporated into growth medium at 100 µM on *E. coli, P. syringae* 4612 and *M. luteus*, respectively. *M. luteus* is shown after 48 h and 7 days of incubation.

In the drop tests, Gram-negative *P. syringae* 4612 cells were inhibited strongly by all thiosulfinates up to 48 h after plating out (Fig. 7). Here, DETS, DMTS, allicin and DBTS appeared more effective than DPTS. Gram-positive *Micrococcus luteus* was inhibited strongly by all thiosulfinates up to 48 hours after plating but after longer incubation growth resumed and after 7 days, for instance, DMTS was hardly different to the control. This result suggests that the effect of DMTS at the test concentration is primarily bacteriostatic rather than bactericidal. For the other thiosulfinates, both bactericidal and bacteriostatic effects were apparent and allicin and DBTS showed the highest antibacterial effects overall (Fig. 7).

### Antifungal activity of thiosulfinates in drop tests

As discussed already, the thiosulfinates seemed to be particularly active against the model fungus *S. cerevisiae*. This is rather fortunate, as a plethora of viable mutants of this eukaryote are available which can be used for chemogenomic profiling studies. Essentially, such studies investigate the divergent sensitivities of the wildtype and different mutants against given compounds and subsequently provide a glimpse into the possible mode(s) of action of those agents^48^. Therefore, this part of the study was designed to test whether the other thiosulfinates might have a similar mode of action to allicin, which is known to target the GSH pool and GSH metabolism^36,38^.

**Figure 8:**
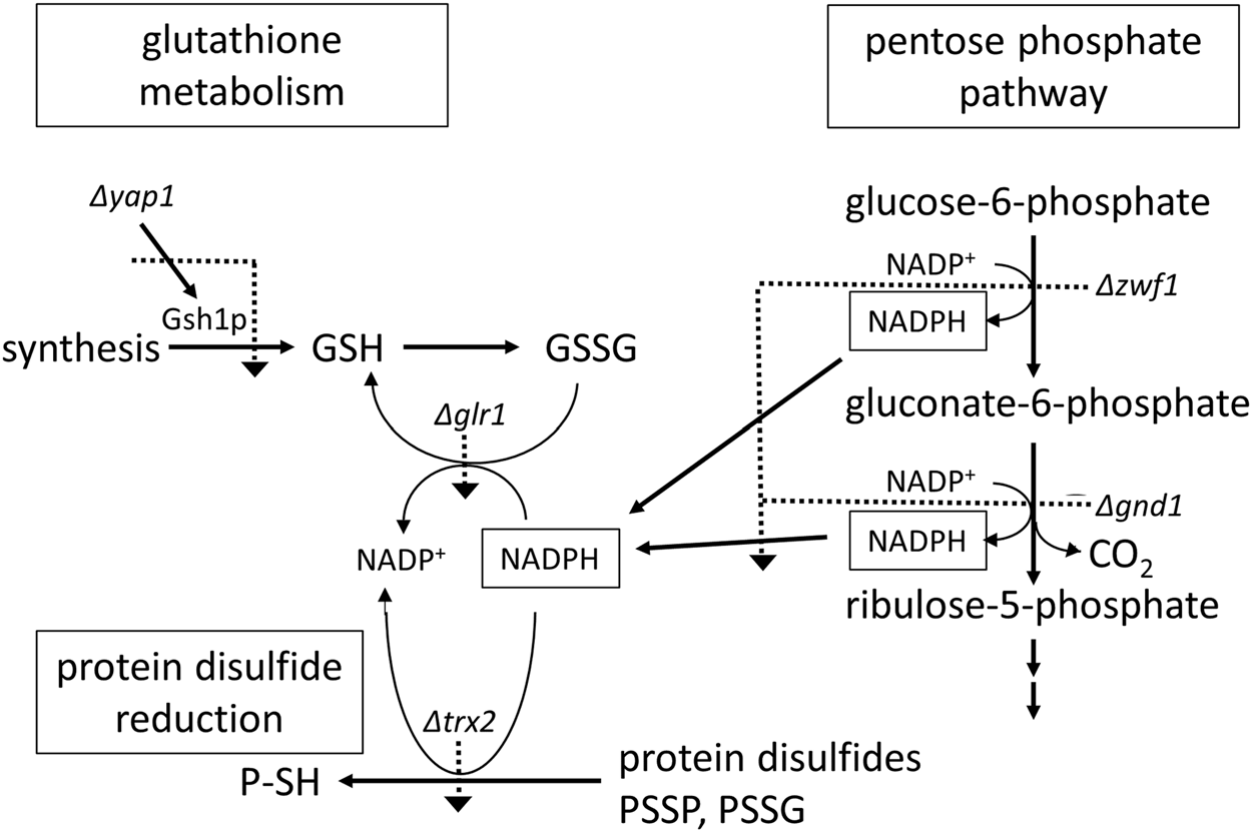
Scheme showing how the various deletion mutants affect GSH synthesis (*Δyap1*) and GSSG reduction, either directly (*Δglr1*), or by blocking the production of NADPH reducing equivalents (*Δzwf1*, *Δgnd1*), or by supressing the NADPH-dependent reduction of protein disulfides (*Δtrx2*, indirectly *Δzwf1*, *Δgnd1*). Dotted lines show the metabolic lesions caused by the deletion mutants.

*Saccharomyces cerevisiae* was used as a model fungus in drop tests on agar medium containing the test substance and in shake culture in 96 well plates (see next section) to assess the antimycotic activity of thiosulfinates. In the drop test, 10 µL of 10-fold serial dilutions were plated out onto control medium, or medium containing 5 µM of the test thiosulfinate. The ability of wildtype (wt) BY4742 cells to grow in the presence of thiosulfinates was compared with the ability of *Δyap1*, *Δglr1*, *Δzwf1*, *Δgnd1*, and *Δtrx2* yeast mutants. Yap1p is a transcription factor that coordinates the oxidative stress response in yeast^49^ and which is activated by direct *S*-thioallylation of specific cysteines by allicin in the C-term of the protein^38^. Yap1p controls the expression of several oxidative stress response genes including *GLR1* and *TRX2*. Glutathione reductase (Glr1p) is an NADPH-dependent enzyme which reduces GSSG back to GSH and Trx2p, which is the major yeast thioredoxin, reduces protein disulfides (PSSP) and glutathiolated proteins (PSSG) back to thiols utilizing thioredoxin reductases that are NADPH-dependent^31^. The major source of NADPH for metabolic reactions in cells is the first two reactions of the oxidative pentose phosphate pathway (PPP), catalysed by glucose-6-phosphate dehydrogenase (Zwf1p) and 6-phosphogluconate dehydrogenase (Gnd1p), respectively. Thus, the mutants chosen are all appropriately relevant for testing and comparing the mechanism of action of allicin in relation to GSH metabolism, with respect to the other thiosulfinates (Fig. 8).

**Figure 9:**
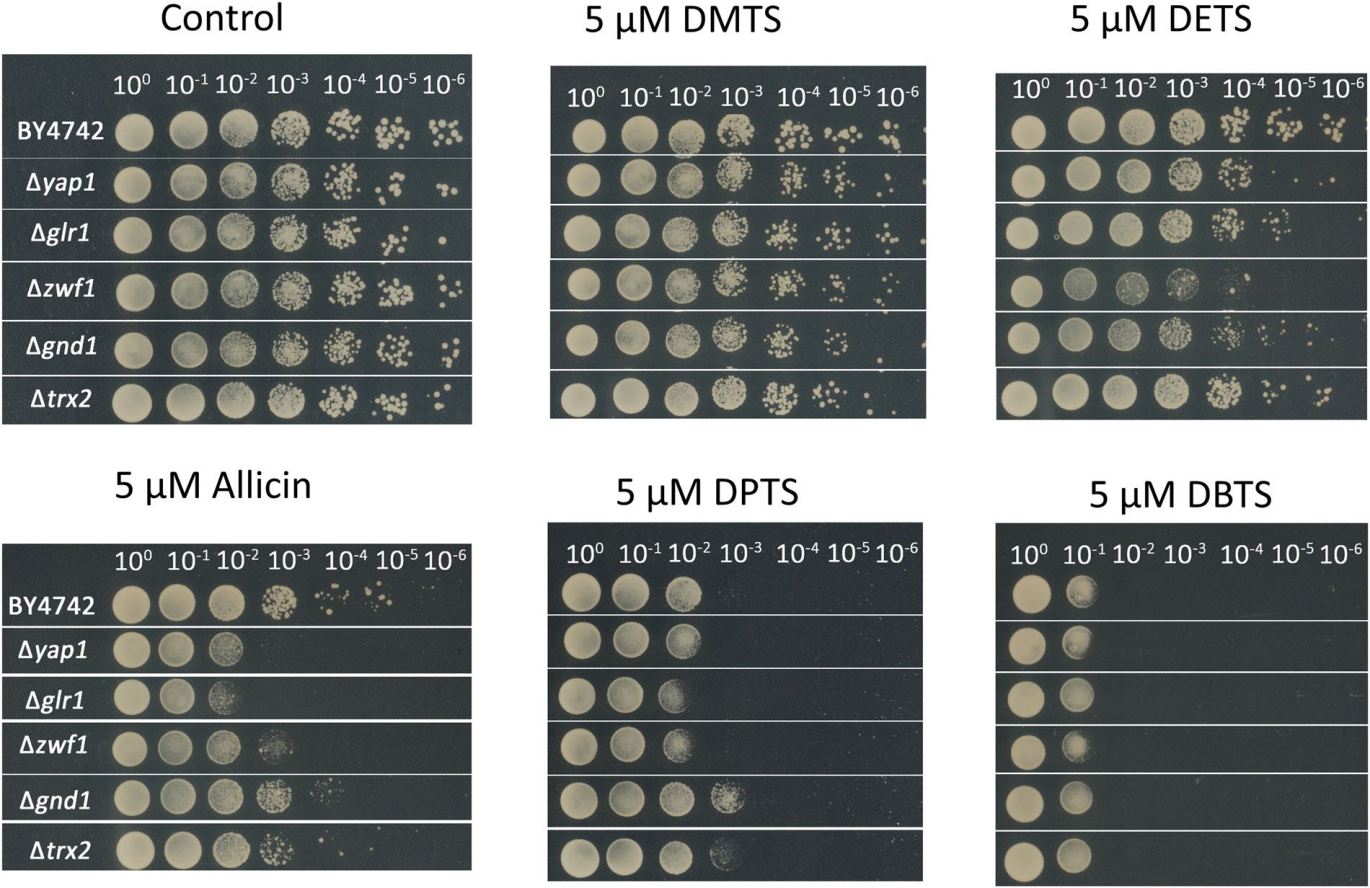
Drop test of *S. cerevisiae* cells on CSM medium containing test thiosulfinates at 5 µM. The wt BY4742 was compared with the *Δyap1* and other mutants. Drops (10 µL) of serial dilutions up to 10^−6^ were plated out. In the control both wt and mutant cells behaved similarly and grew down to the 10^−6^ dilution. The effects of the various thiosulfinates are shown in the remaining panels.

**Figure 10:**
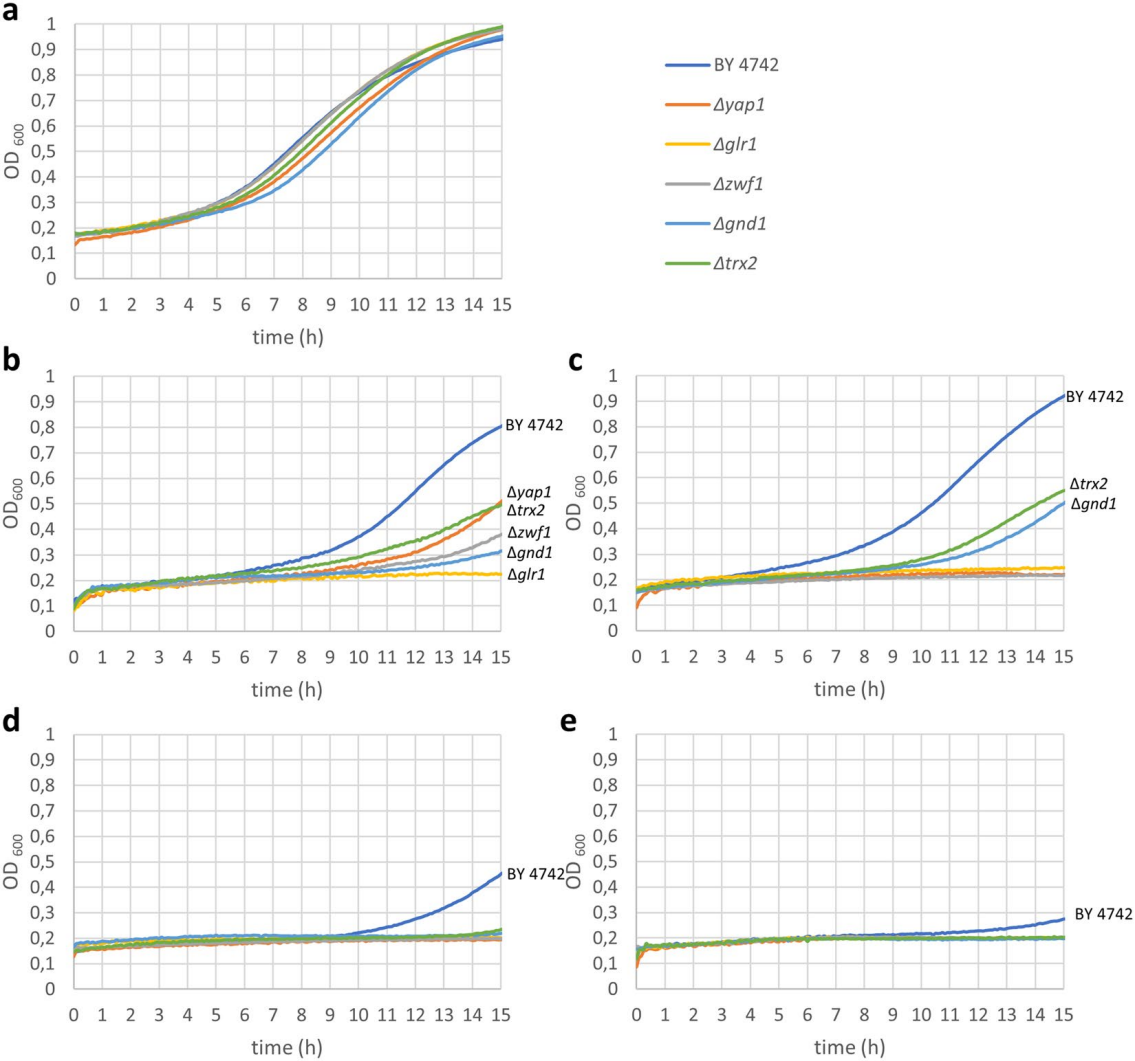
Effects of 50 µM thiosulfinates on the growth in shake culture of wt BY4742 and *Δyap1*, *Δglr1*, *Δzwf1*, *Δgnd1*, and *Δtrx2* mutant yeast cells in CSM. (**a**) CSM alone (control); (**b**) DMTS; (**c**) DETS; (**d**) DATS (allicin); (**e**) DPTS. The experiments were repeated twice with similar results.

**Figure 11:**
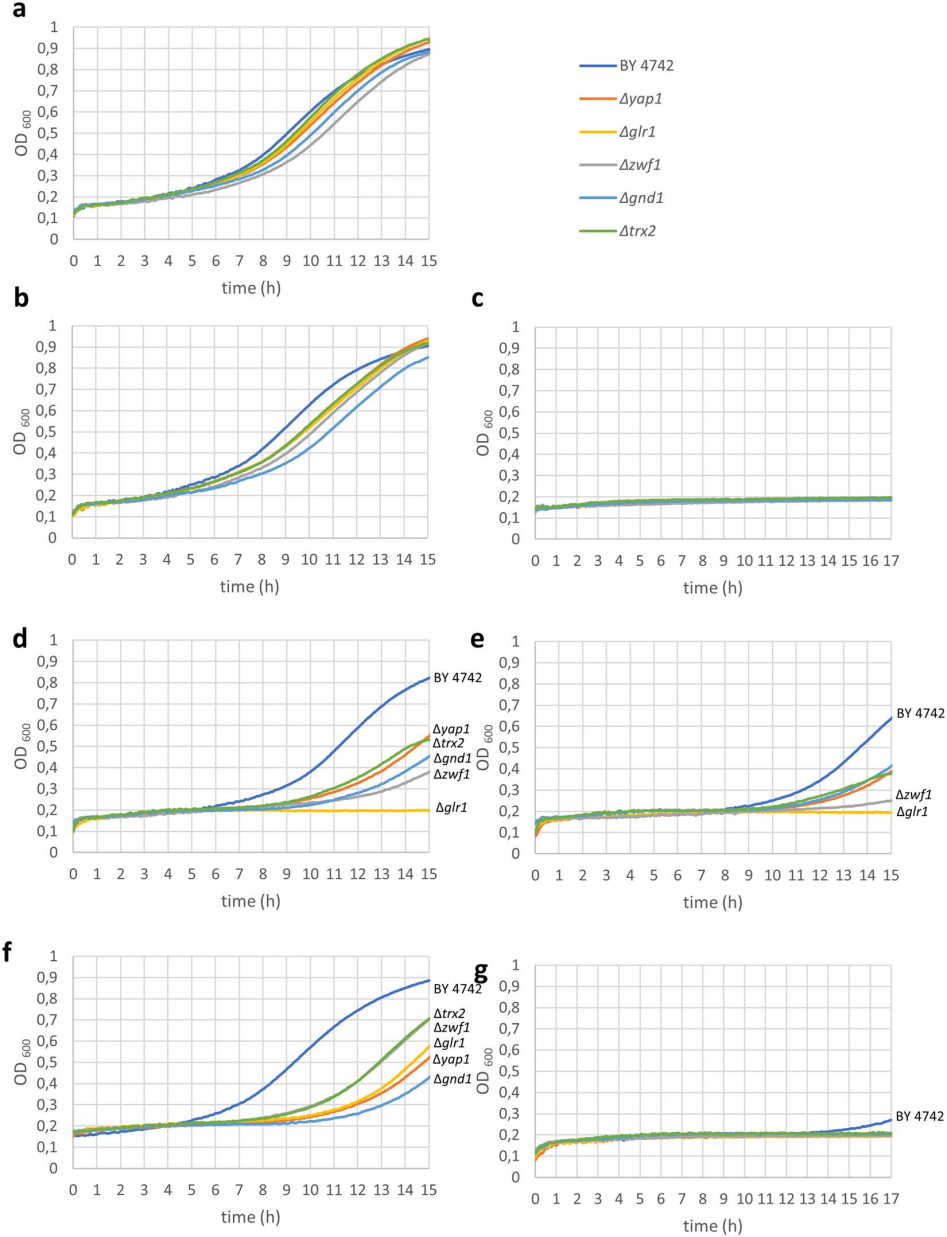
Effect of DBTS on the growth in CSM of wt BY4742 and *Δyap1*, *Δglr1*, *Δzwf1*, *Δgnd1*, and *Δtrx2* mutant yeast cells in CSM and the synergistic effect of DMF with allicin. (**a**,**b**) are controls in the absence and presence of 0.5% DMF. (**c**) 5 µM; (**d**) 10 µM and (**e**) 25 µM DBTS in the presence of 0.5% DMF, respectively. (**f**) 25 µM allicin and (**g**) 25 µM allicin in 0.5% DMF. The experiments were repeated twice with similar results.

In the absence of thiosulfinate in the medium the wt and mutant yeast strains grew equally well down to the 10^−6^-fold dilution. DMTS at 5 µM did not affect the growth of the wt or any of the mutant cells (Fig. 9). DETS at 5 µM was not inhibitory for BY4742 wildtype, but clearly inhibited *Δyap1*, *Δglr1*, *Δzwf1*, *Δgnd1* cells at this concentration. Allicin was the first compound in the series that inhibited the wildtype at 5 µM in addition to showing a greater inhibition in *Δyap1 Δglr1*, *Δzwf1*, *Δgnd1* and marginally in *Δtrx2* cells. Interestingly, the drop test did not resolve any differential toxicity for DPTS at 5 µM between the wt and most mutants. However, see Fig. 10 in the next section with respect to growth kinetics. DBTS showed the highest toxicity to yeast of the thiosulfinates tested and again the drop test did not resolve any differential toxicity for DPTS at 5 µM between the wt and the mutants. However, see Fig. 11 in the next section with respect to growth kinetics.

These findings, albeit of a preliminary nature, are rather intriguing as they (a) confirm the considerable toxicity of the various thiosulfinates towards yeast and perhaps fungi in general, (b) support the overall impression that allicin, DPTS and DBTS are more active compared to the shorter chain analogues and (c) also point towards similar underlying mode(s) of action against which GSH metabolism plays an important role. As mentioned in the introduction, induction of oxidative stress is probably just one aspect of antimicrobial activity, and it would not be surprising if some of the test compounds had additional mechanisms. For instance, highly toxic benzylthiol may be formed as part of DBTS intracellular redox transformations.

### Antifungal activity of thiosulfinates in shake culture

As is apparent from the results of the antimicrobial assays described so far, the relative sensitivity of an organism to an antibiotic is test-dependent and threshold inhibitory concentrations can vary depending on the conditions. Furthermore, the ability of a particular test to resolve different sensitivities between isolates also varies. Therefore, the effects of thiosulfinates on wt and *Δyap1*, *Δglr1*, *Δzwf1*, *Δgnd1*, and *Δtrx2* yeast mutants were also investigated in shake culture because this has the additional advantage of providing relative growth kinetics and not just an end-point result^48^. In contrast to the stationary culture conditions in the MIC and MBC tests, or in drop tests where cells are plated onto medium containing the test substance, in shake culture cells tend to grow more robustly and generally tolerate higher concentrations of antibiotics. Previous experiments had shown that 50 µM allicin reduced the growth rate of wt BY4742 cells by approximately 50% at the end time point of 15 h in shake culture in CSM medium. Therefore, in these experiments we exposed the cells to 50 µM of thiosulfinates for comparison.

The growth of BY4742 wildtype and the mutants in CSM medium controls was similar, showing no variation in the timing of the start of exponential growth, the rate of growth or end point cell titre reached after 15 h. Figure 10a shows a representative plot chosen from 4 replicates.

DMTS at 50 µM led to a delay of a few hours in the start of exponential growth of the wt but in contrast to the drop test (Fig. 8) the effect on the mutants was very pronounced, with longer lag phases until the resumption of growth and a much-reduced cell titre after 15 h. Furthermore, at 50 µM DMTS completely inhibited growth of the *Δglr1* mutant (Fig. 10b).

DETS at 50 µM led to a slight delay in the start of exponential growth of the wt but completely inhibited growth of the *Δyap1*, *Δglr1*, *Δzwf1* mutants up to the end of the experiment (Fig. 10c).

In contrast to DMTS and DETS, allicin at 50 µM caused a marked inhibition of the wt, delaying the start of exponential growth and leading to a much-reduced endpoint cell titre. Growth of all of the mutants was completely inhibited up to the end of the experiment (Fig. 10d).

DPTS at 50 µM was more inhibitory than allicin to the wt and again completely inhibitory to all of the mutants (Fig. 10e).

Taken together the results of the shake culture experiments show a gradient of increasing inhibitory activity from DMTS < DETS < allicin < DPTS, corresponding to the increasing log*P* along the thiosulfinate series. This might be coupled with the relative ease with which the compounds can traverse the membrane to gain access to the cells. Allicin, for instance (log*P* = 1.35), is readily membrane permeable and indeed causes transient pore formation in biological and artificial membranes^40,41^. This activity series can also be seen in the drop test, but the increased sensitivity of the mutants to DPTS compared to the wt was not resolved (Fig. 9).

Because DBTS is insoluble in water, it was dissolved in dimethyl formamide (DMF) which was present at 0.5% v/v in the final test solutions. DMF at this concentration showed no significant effect on growth of either the wt or the mutants in comparison to the CSM controls (Fig. 11a,b). However, a dose-dependent inhibition of wt and mutants can be seen at 5, 10 and 25 µM, with the latter concentration being completely inhibitory to the growth of all yeast strains (Fig. 11c–e). It can clearly be seen that the *Δglr1* mutant is the most sensitive and this was completely inhibited for the duration of the experiment at 5 µM DBTS in 0.5% DMF. This very high degree of inhibition compared to the other thiosulfinates, suggests that DBTS had the greatest activity of the thiosulfinate series against *Saccharomyces cerevisiae*, a result which corresponds to the drop test results shown in Fig. 9. Yet this result must be viewed with caution. Notably, a synergistic effect was observed between DMF and allicin, which at 25 µM showed a lesser inhibitory effect without DMF than with 0.5% DMF where it also completely inhibited the growth of wt and all mutant cells (Fig. 11f,g). Whilst DMF alone had no effect on cell growth, there was most likely also a synergistic effect between DBTS and DMF. These findings caution against the often naïve use of common solvents such as DMF or DMSO to enhance the solubility of refractory test substances.

To sum up the results of the chemogenetic profiling, the observation that the chosen mutants were generally more sensitive to thiosulfinates than the wt suggests that the other thiosulfinates are probably acting similarly to allicin and targeting the cellular GSH pool and GSH metabolism as well as resulting in protein thiol oxidation^3,20,36,38^. The strongly susceptible phenotype of the *Δglr1* mutant in comparison to the weaker susceptibility of the *Δtrx2* mutant is particularly informative in this regard. Thus, it seems that GSH is the first line of cellular defence and the ability of the cells to reduce GSSG to GSH is crucial for the cells’s resistance to allicin and the other thiosulfinates. Oxidizing accessible protein thiols to PSSP and PSSG is also part of allicin’s mode of action^36^ and this is reflected in the sensitivity of the *Δtrx2* mutant, a trait also shown for the analogue thiosulfinates and thus supporting a similar mode of action as for allicin (Figs. 9–11). The importance of Zwf1p and Gnd1p activities is clear because these enzymes are the major source of NADPH to provide reducing potential needed for Glr1p to reduce GSSG, and Trx2p activity via NADPH-dependent thioredoxin reductases.

### Effect of thiosulfinates on the viability of human lung epithelial carcinoma cells (A549)

If an antibiotic is to be used in the treatment of patients, then the differential susceptibility of mammalian cells and target pathogen cells should be as high as possible. Allicin is a biocide and kills mammalian cells as well as bacteria and fungi in a dose-dependent manner. The effects of allicin on a range of mammalian cell lines has been investigated^29^, but the mammalian cell toxicity of the other thiosulfinates is unknown. In the work reported here, an MTT test for cell viability with human alveolar basal epithelial adenocarcinoma (A549) cells was performed. However, because allicin reduces the adherence of cultured cells, and this leads to variable losses during the usual washing procedure, any unreacted thiosulfinate was titrated out by adding excess cysteine. Cysteine itself, added to medium without cells, did not lead to a reduction of MTT.

**Figure 12:**
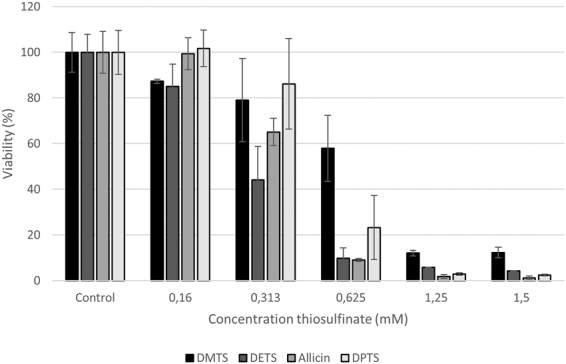
Dose-dependent effect of thiosulfinates on the viability of cultured human A549 cells. The experiment was repeated 4 times (n = 4–8) with similar results and a set of representative data is shown.

All the thiosulfinates caused a dose-dependent decrease in the viability of cultured human A549 cells, pivotal over the 0.625–1.25 mM range (Fig. 12). There was some variation in the relative activities of different thiosulfinates but these were not consistently statistically significant between experiments. Nevertheless, the tendency that DMTS was least toxic and allicin most toxic to A549 cells was a clearly visible trend in all the experiments and the data confirm that the analogues are of similar toxicity to allicin. Therefore, like allicin, because of a relatively low differential toxicity between bacteria and mammalian cells, thiosulfinates might be better used at low concentration in combination with other clinically proven antibiotics, for example against MDR strains where allicin has generally been shown to be effective^25^. Because thiosulfinates are titrated out by GSH, oral use is in any case likely to be precluded because it will not be possible to achieve therapeutic concentrations via the oral route^50^. This is clear because a single clove of garlic can produce up to 5 mg of allicin, a substance toxic to cells in µg amounts, and yet, garlic is consumed worldwide without detrimental effects to the consumer. In this regard, specialist applications must be considered. Thus, the shortage of volatile antibiotics, coupled with reports from the pre-antibiotic era of successful treatment of tuberculosis patients by garlic vapour inhalation^29,30^, indicate such a potential and emphasize the fact that cells in suspension culture do not have the same environment as cells in the body. In the intact organism, with a circulating blood supply and continual replenishment of GSH, particularly in the lungs which are continually exposed to oxidative stress, the volatile thiosulfinates may be able to play a role against lung-pathogenic organisms via the direct pulmonary route, either alone or in combination with conventional antibiotics taken orally^25^. This important point needs addressing in the future in long-term animal studies, which are beyond the scope of the present study.

### Effect of thiosulfinates on the viability of tobacco bright-yellow-2 (BY-2) cells and Arabidopsis seedling root growth

We have previously shown that although allicin traverses biological and artificial membranes easily^41^, it does not penetrate the wax plates of the plant cuticle efficiently and when sprayed onto leaves up to 2.4 mM showed no plant toxicity, making it a good contact fungicide, comparable in effectivity to the commercial fungicide Cuprozin^TM^ against cucumber downy mildew^51^. Furthermore, allicin was shown to be as effective as the commercial fungicide Aatiram^®^ in sanitizing carrot seed infested with *Alternaria* spp. and may be suitable for controlling other seed-borne diseases^52^. However, young seedling roots do not have a cuticle and we have previously shown that allicin over the 25–100 µM range progressively inhibited root growth in *Arabidopsis* seedlings and at > 500 µM caused extensive bleaching of the cotyledons^3^. Plant toxicity data for the other thiosulfinates is lacking, therefore we tested the effect of a one hour exposure to thiosulfinates on the viability of tobacco bright yellow 2 (BY-2) cell cultures^53^, and the effect on *Arabidopsis* root growth.

**Figure 13:**
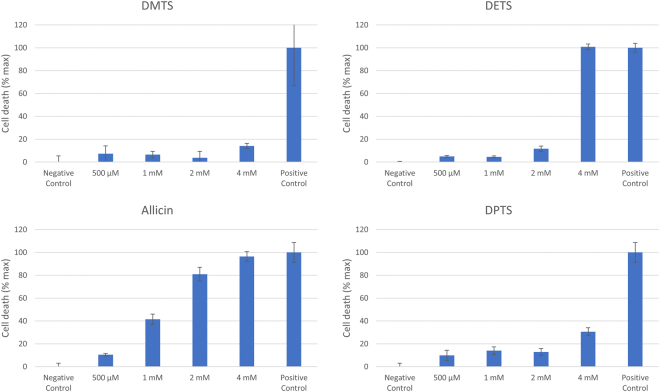
Effect of thiosulfinates on the viability of tobacco BY-2 cells in shake culture. Cells were exposed to the stated thiosulfinate concentration for one hour, stained with Evans blue, and bound dye measured at A_600_.

Evans Blue was used to stain dead BY-2 cells^54^ and the trend observed with A549 adenocarcinoma cells, that DMTS was least toxic and DATS most toxic, was clearly reiterated (Fig. 13).

We also compared the effect of thiosulfinates on the growth of *Arabidopsis* seedling roots. Seeds were allowed to germinate for three days before placing on medium containing thiosulfinate. Root length was measured after three days of continual exposure. *Arabidopsis* Col-0 wt and *pad2* and *gr1* mutants in the Col-0 background were tested. The *pad2* line is mutated in the glutamate cysteine ligase gene and has only approximately 20% of the GSH level found in the wt^55^. The *gr1* mutant line is a knockout mutant of glutathione reductase and has a higher proportion of GSSG in the glutathione pool because it cannot reduce GSSG back to GSH^56^.

**Figure 14:**
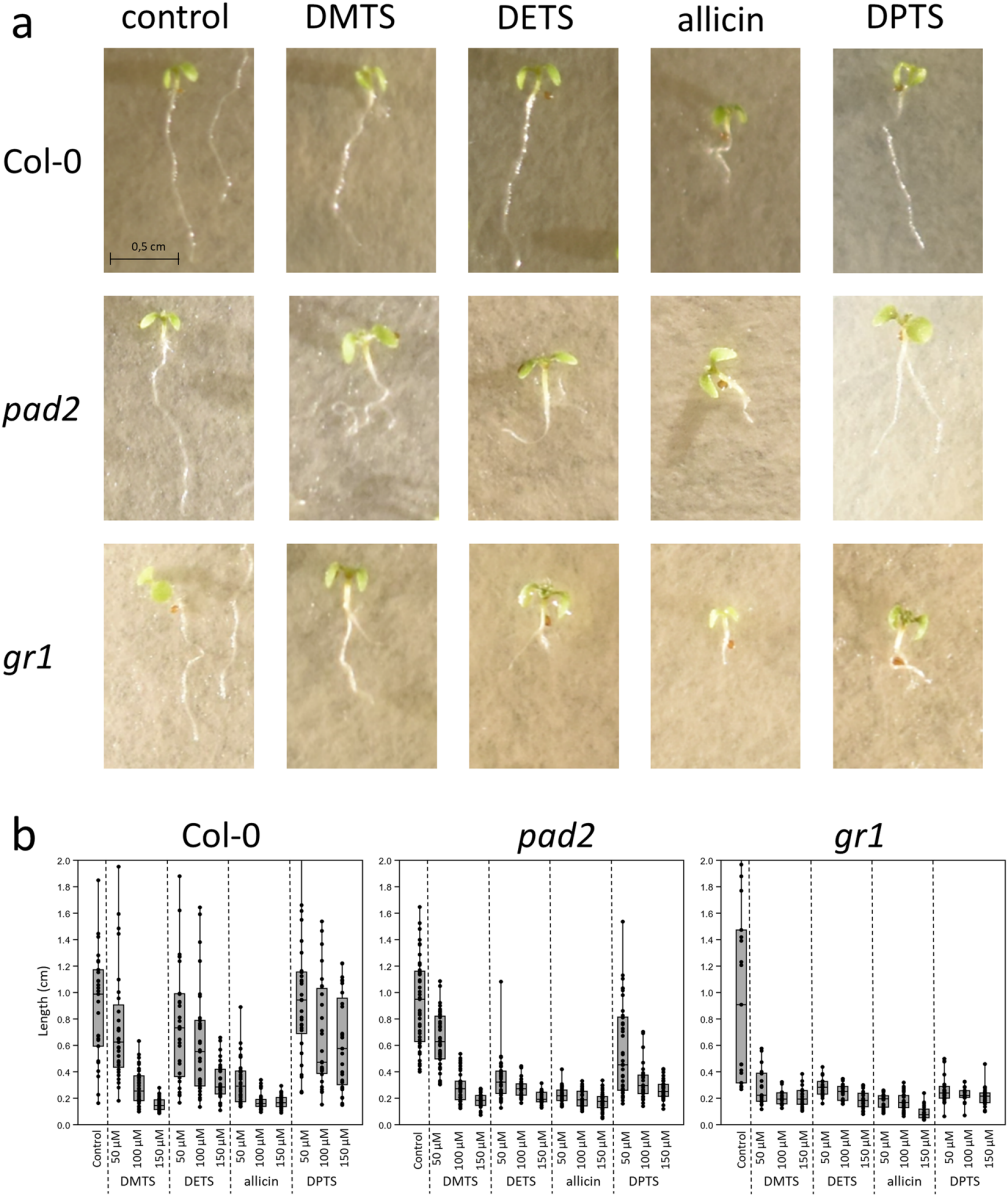
Effect of thiosulfinates on *Arabidopsis* root growth. (**a**) shows the phenotypes of Col-0 (wt), *pad2* and *gr1* seedlings observed after three days of continual exposure to 50 µM thiosulfinate in the growth medium. Scale bar = 5 mm. (**b**) shows the effect of continual exposure to 50, 100 and 150 µM thiosulfinate on root growth of Col-0 (wt), *pad2* and *gr1* seedlings. The box plots show the range of individual measurements, the mean +/− standard deviation, and the median value indicated as a horizontal line.

As can be seen in Fig. 14a, the mutants *pad2* and *gr1*, compromised respectively in GSH synthesis and GSSG reduction, are more sensitive to thiosulfinate treatment than the Col-0 wt. Root growth was impaired and, interestingly, approximately 30% of the mutant seedlings exposed to 50 µM DPTS showed a branched root phenotype. This was not observed in the wt and this phenomenon may be worthy of further investigation. The concentration-dependent inhibition of root growth by thiosulfinates is shown in Fig. 14b. The enhanced sensitivity of GSH metabolism mutants compared to the Col-0 wt confirms the results of the chemogenetic screen with yeast mutants (Figs. 9–11) and again points to the central role of cellular GSH in the resistance of cells to thiosulfinates.

## Materials and Methods

### Materials

MSDS was purchased from Acros Chemicals (Geel, Belgium). DADS (80%), DEDS, DPDS and DBDS were purchased from Sigma Aldrich (Munich, Germany). Formic acid (98%, p.a.) was purchased from Carl Roth (Karlsruhe, Germany). H_2_O_2_ (30%) was purchased from Merck (Darmstadt, Germany). Acetic acid (100%, p.a.) was purchased from Carl Roth. TLC was performed using Merck TLC Silica gel 60 F254 with concentration zone. Solvent A (n-hexane 99% p.s.) was purchased from Carl Roth. Solvent B (ethyl acetate 99.5% p.s.) was purchased from Carl Roth. Liquid chromatography was performed using silica gel 60 (0.04–0.063 mm (230–400 mesh)) purchased from Carl Roth.

The synthetic procedure for thiosulfinates was after Albrecht *et al*.^32^.

### Synthesis of Dimethylthiosulfinate (DMTS)

Dimethyldisulfide (1.3 g; 13.8 mmol) was dissolved in 5 mL formic acid and stirred for 5 minutes on ice. H_2_O_2_ (30%; 2.4 mL; 23.7 mmol) was added slowly to the mixture. HPLC and TLC confirmed the reaction had reached completion after 90 minutes. The reaction was stopped by addition of 25 mL H_2_O. The mixture was extracted 3 times with 30 mL dichloromethane (DCM) and the organic layer was washed with saturated Na_2_CO_3_ solution until the acid was completely neutralized. The organic layer was separated, dried with Na_2_SO_4_ and filtered. The solvent was evaporated under reduced pressure at room temperature to yield a clear oily substance of characteristic smell. Yield: 1.44 g; 13 mmol. DMTS synthesis was stoichiometric, yielding a pure product with a single HPLC peak and no further purification was carried out.

$${}^{1}{\rm{H}}\,{\rm{N}}{\rm{M}}{\rm{R}}\,(500\,{\rm{M}}{\rm{H}}{\rm{z}},{{\rm{C}}{\rm{D}}{\rm{C}}{\rm{l}}}_{3})\,\delta \,3.29\,(s,3{\rm{H}}),\delta \,2.69(s,3{\rm{H}})$$

$${}^{13}\,{\rm{C}}{\rm{NMR}}\,(125\,{\rm{MHz}},\,{{\rm{CDCl}}}_{3})\,{\rm{\delta }}\,18.3,\,48.9$$

### Synthesis of Diethylthiosulfinate (DETS)

Diethyldisulfide (2.07 g, 16.96 mmol) was dissolved in 5 mL formic acid and stirred on ice for 5 minutes. H_2_O_2_ (3 mL) was slowly added to the mixture. HPLC analysis indicated the reaction had gone to completion after 90 minutes. The reaction was stopped by addition of 25 mL H_2_O and the organic compounds were extracted by washing the mixture 2 times with 10 mL DCM. The solvent was evaporated under reduced pressure at room temperature and the residue was dissolved in an ethyl acetate: n-hexane mixture (1:2). The product was purified on a silica gel 60 column with the same mixture used as mobile phase. The product-containing fractions were combined, and the solvents were removed by rotary evaporation at room temperature. The product was a clear oil with a pungent smell. Yield: 1.7 g; 12.3 mmol

$${}^{1}{\rm{H}}\,{\rm{N}}{\rm{M}}{\rm{R}}\,(500\,{\rm{M}}{\rm{H}}{\rm{z}},\,{{\rm{C}}{\rm{D}}{\rm{C}}{\rm{l}}}_{3})\,\delta \,1.34-1.37\,({\rm{t}},3{\rm{H}}),\,\delta \,1.39-1.43\,({\rm{t}},3{\rm{H}}),\delta \,3.03-3.17\,({\rm{m}},4{\rm{H}})$$

$${}^{13}{\rm{C}}{\rm{NMR}}\,(125\,{\rm{MHz}},\,{{\rm{CDCl}}}_{3})\,{\rm{\delta }}\,7.7,16.1,\,26.9,49.8$$

### Synthesis of Dipropylthiosulfinate (DPTS)

Dipropyldisulfide (2.07 g, 13.8 mmol) was dissolved in 5 mL formic acid and stirred on ice for 5 minutes. H_2_O_2_ (30%, 3 mL) was slowly added to the mixture. The reaction was complete after 4 hours. To stop the reaction 25 mL of H_2_O were added and the organic components were extracted by washing 3 times with DCM. The combined organic phases were washed with saturated Na_2_CO_3_ solution, dried over Na_2_SO_4_ and filtered. The solvent was removed under reduced pressure at room temperature to yield a clear oil. Yield: 2.26 g; 13.6 mmol. DPTS synthesis was stoichiometric, yielding a pure product with a single HPLC peak and no further purification was carried out.

$${}^{1}{\rm{H}}\,{\rm{N}}{\rm{M}}{\rm{R}}\,(500{\rm{M}}{\rm{H}}{\rm{z}},\,{{\rm{C}}{\rm{D}}{\rm{C}}{\rm{l}}}_{3})\,\delta \,0.99-1.10\,({\rm{m}},\,6{\rm{H}}),\delta \,1.75-1.88\,(m,4{\rm{H}}),\,\delta \,3.02-3.16\,({\rm{m}},4{\rm{H}})$$

$${}^{13}{\rm{C}}\,{\rm{N}}{\rm{M}}{\rm{R}}\,(125\,{\rm{M}}{\rm{H}}{\rm{z}},{{\rm{C}}{\rm{D}}{\rm{C}}{\rm{l}}}_{3})\,\delta \,13.2,17.2,\,24.3,34.9,58.0$$

### Synthesis of di-2-propenethiosulfinate (Allicin)

Diallyldisulfide (DADS; 2 g, 13.7 mmol) was mixed in 5 mL formic acid and stirred for 5 minutes at 0 °C. H_2_O_2_ (30%; 3 mL, 29.6 mmol) was added slowly to the mixture. The reaction was stopped by addition of 25 mL H_2_O after approx. 4 hours and the mixture was extracted 3 times with DCM. The solvent was removed under reduced pressure and the product was dissolved in the eluent, a mixture of n-hexane and ethyl acetate (2:1). Separation was performed *via* liquid chromatography using 150 mm silica gel 60 in a column with a diameter of 30 mm. Fractions were collected into tubes cooled in an ice bath and TLC was used to identify fractions containing only allicin. Those fractions were combined, dried with anhydrous sulfates (e.g. Na_2_SO_4_, MgSO_4_ or CuSO_4_) and filtered. The solvents were removed under reduced pressure at RT to yield a clear, oily substance that smells like garlic. Yield: 1.64 g, 10.1 mmol, 73.7%.

$${}^{1}{\rm{H}}\,{\rm{N}}{\rm{M}}{\rm{R}}\,(500\,{\rm{M}}{\rm{H}}{\rm{z}},{{\rm{C}}{\rm{D}}{\rm{C}}{\rm{l}}}_{3})\,\delta \,3.70-3.75\,({\rm{m}},\,4{\rm{H}})\,\delta \,5.14-5.42\,({\rm{m}},4{\rm{H}})\,\delta \,5.68-5.88\,({\rm{m}},\,2{\rm{H}})$$

$${}^{13}{\rm{C}}\,{\rm{N}}{\rm{M}}{\rm{R}}\,(125\,{\rm{M}}{\rm{H}}{\rm{z}},{{\rm{C}}{\rm{D}}{\rm{C}}{\rm{l}}}_{3})\,\delta \,35.1,\,59.8,\,119.1,\,124.1,125.8,132.9$$

### Synthesis of Dibenzylthiosulfinate (DBTS)

Dibenzyldisulfide (0.51 g, 2.03 mmol) was suspended in 5 mL acetic acid. Cold H_2_O_2_ (0.23 g, 30%) was added dropwise to obtain an equimolar concentration of the reactants. After 5 h the mixture was extracted two times with DCM, the organic phases combined, and the solvent removed under reduced pressure to yield a white solid substance. The substance was dissolved in a mixture of ethyl acetate and n-hexane (1:2) and purified on a silica gel column. The product-containing fractions were combined, and the solvents removed under reduced pressure at RT. The residual white powder was characterized by NMR as dibenzylthiosulfinate. Yield: 0.15 g, 0.58 mmol.

$${}^{1}{\rm{H}}\,{\rm{N}}{\rm{M}}{\rm{R}}\,(500\,{\rm{M}}{\rm{H}}{\rm{z}},{{\rm{C}}{\rm{D}}{\rm{C}}{\rm{l}}}_{3})\,\delta \,4.15-4.28\,({\rm{m}},\,4{\rm{H}})\,\delta \,7.12-7.36\,({\rm{m}},\,10{\rm{H}})$$

$${}^{13}{\rm{C}}\,{\rm{N}}{\rm{M}}{\rm{R}}\,(125\,{\rm{M}}{\rm{H}}{\rm{z}},\,{{\rm{C}}{\rm{D}}{\rm{C}}{\rm{l}}}_{3})\,\delta \,36.1,\,62.2,\,127.7,\,128.7,\,128.8,\,129.1,\,129.9,\,130.3,\,136.6$$

### Analysis of thiosulfinates by HPLC

A Jasco HPLC system with a UV-2077 multichannel detector and a PU-980 pump was used. The separation was performed on a Prontosil Kromaplus column (150 × 4.6 mm, 5 µm) at 25 °C and the UV detector was operated at 254 nm. The flow rate was 1 mL min^−1^. The mobile phase consisted of H_2_O (A) and methanol. The following gradient was used: 56% A (0 min) 53% A (10 min) 7% A (15 min) 7% A (30 min) 56% A (31 min) 56% (35 min).

### Thermal stability of thiosulfinates

Aqueous solutions (500 µL, 10 mM) of DMTS, DETS, DPTS, and allicin were heated in sealed tubes in a thermostated shaker for 10 minutes at 99 °C and cooled on ice afterwards. A 10 mM DBTS solution in aqueous 10% DMF was treated similarly. The chemical decomposition of the compounds was analysed with HPLC using UV detection.

### Test microorganisms used in this study

*E. coli* K12, *Pseudomonas fluorescens* and *Pseudomonas syringae* pv. *phaseolicola* 4612 were used as representative Gram-negative bacteria. *E. coli* was grown on LB-medium (10 g L^−1^ tryptone, 5 g L^−1^ yeast extract, 5 g L^−1^ NaCl, 15 g L^−1^ agar for solid medium, all chemicals from Carl Roth, Karlsruhe) without selection and incubated at 37 °C. *Pseudomonas* isolates were grown at 28 °C on King’s B medium (20 g L^−1^ peptone, 12.6 g L^−1^ glycerol, 1.5 g K_2_HPO_4_ (anhydrous); upon autoclaving, 1.5 g L^−1^ MgSO_4_ (heptahydrate) was added. For solid medium, 15 g L^−1^ agar was added; all chemicals were purchased from Carl Roth, Karlsruhe, Germany).

### *Micrococcus luteus* (*Ml*), grown on LB-medium at 28 °C, was used as a representative Gram-positive bacterium

The haploid *Saccharomyces cerevisiae* yeast strain BY4742 (Matα; his3Δ1;leu2Δ0, lys2Δ0, ura3Δ0) was used as a model fungus in tests with different thiosulfinates. The BY4742 mutant Δ*yap1* (YML007w) used in this study lacks a redox-sensitive transcription factor that is important for oxidative stress response. All mutants were obtained from the EUROSCARF Collection, University of Frankfurt (Main), Germany (http://www.euroscarf.de/).

Yeast was grown in CSM medium (0.79 g L^−1^ CSM Drop-Out: Complete [ForMedium, Norwich, United Kingdom]; 6.9 g/l Yeast Nitrogen Base [ForMedium, Norwich, United Kingdom]; 40 g L^−1^ D-Glucose [Carl Roth, Karlsruhe, Germany], 15 g L^−1^ agar for solid medium.

### Determination of minimal inhibitory concentration (MIC) and minimal bacteriocidal (MBC) concentration

Susceptibility testing was performed following European Committee on Antimicrobial Susceptibility Testing (EUCAST) guidelines using the broth dilution method in 96-well microtiter plate format^47^. Bacteria (*Escherichia coli*, *Pseudomonas syringae* pv. *phaseolicola* 4612, *Pseudomonas fluorescens Pf-*01, *Micrococcus luteus*) were grown over-night in media and temperatures as described above. The flasks were shaken at 220 rpm. At the next day, the bacteria were diluted to an OD_600_ of 0.3 and used in a dilution of 1:234. The thiosulfinates were dissolved in deionized water to a final concentration of 2,048 µg mL^−1^. In a 96-well plate, a 2-power dilution series was performed in 50 µL volume and 50 µL of the diluted bacteria culture were added. The concentrations varied therefore from 1024 µg mL^−1^ to 1 µg mL^−1^ and one well was reserved for the control. The microtiter plates were incubated for 20 h at the optimal temperatures for the bacteria, as mentioned above. The lowest concentrations that showed no visible growth of bacteria is the minimal inhibitory concentration (MIC).

To determine the minimal bactericidal concentration, 10 µL out of each well was inoculated onto a plate with the optimal medium for the particular bacterium and incubated for 24 h. The lowest concentrations that showed no growth after 24 h gave the MBC value.

### Plate inhibition zone assay to test for antimicrobial activity against bacteria and yeast

An overnight culture of the bacteria grown in LB-medium under optimal conditions (*E. coli*: 220 rpm, 37 °C, other bacteria: 220 rpm, 28 °C) was diluted to about OD_600_ = 0.1 and further grown to OD_600_ = 0.5. Temperature of LB-agar was equilibrated in the water bath to 50 °C. 50 µL of bacteria suspension is added to 10 mL of medium, mixed in a falcon tube and poured into a petri-dish with 9 cm diameter. Upon solidification holes were punched out using a cork borer (6 mm diameter). Each hole was filled with 20 µL of thiosulfinate solution (8 mM, 4 mM and 2 mM). Plates were grown for 20 h. The diameter of the inhibition zone was measured and plates were photographed.

An overnight culture of yeast in CSM medium was diluted to about OD_600_ = 0.1 and grown again at 28 °C and 220 rpm to OD_600_ = 0.5. 50 µL were added to 10 mL of 50 °C warm CSM medium and mixed in a falcon tube. Further procedure was as with bacteria (see above).

### Antimicrobial effects of thiosulfinates via the gas phase

Bacteria were seeded into LB-medium as described above. The different thiosulfinates were diluted with water to a final concentration of 80 mM. DBTS was first solved in DMF and subsequently also diluted to 80 mM with a final concentration of 5% DMF. Drops of 20 µL were placed in the center of the Petri-dishes lid. The Petri dish was incubated upside down over night at the particular temperature (*E. coli* 37 °C, other bacteria 28 °C). For a time resolved test the droplet was removed after following hours passed: 0.25, 0.5, 0.75, 1, 1.5, 2, 3, 4, 20. The plates were incubated for 20 hours at 37 °C.

### Drop-test using yeast wildtype and mutant strains

A yeast-overnight culture in CSM (see above) was diluted to OD_600_ = 0.8. Solid CSM medium was equilibrated to a temperature of 50 °C and thiosulfinates were added to a final concentration of 5 µM. Medium was poured in a quadrangular Petri-dish (10 × 10 × 2 cm). Dilutions (10 µL) of the yeast suspension (10^0^–10–7 in medium) were spotted onto the solid medium and plates were incubated at 28 °C for 44 h. It was optically evaluated to which dilution yeast was able to grow.

### Yeast growth kinetics

The compounds tested were diluted in CSM medium to final concentrations as indicated in the figure. A yeast overnight-culture was adjusted to OD_600_ = 0.9. 158 µL medium containing were mixed with 7 µL diluted culture per well. Growth was monitored in a plate reader (Berthold TriStar^2^S LB 942) at 600 nm over a time period of 15 h at 28 °C and shaking.

### MTT test for viability of human cells

Human lung epithelial carcinoma cells (A549), a cell line developed in 1972 and widely used as an *in vitro* model (ATCC-CLL 185), were incubated for 2 weeks at 37 °C and 5% atmospheric CO_2_ in 96-well plates. Cells were cultivated in 100 µL DMEM medium with penicillin/streptomycin 1% (v/v) (each 10,000 U/mL, Lonza, Verviers, Belgium) and fetal bovine serum (FBS) 10% (v/v) (Sigma-Aldrich, St. Louis, USA). After removal of the medium by aspiration, cells were exposed to thiosulfinates (dissolved in medium) for 1 h (controls with medium only). Thiosulfinate concentrations between 0.16 and 2.5 mM (two-power dilution series) were tested. After incubation, unreacted thiosulfinate was titrated out by the addition of 100 µL 6 mM cysteine (AppliChem GmbH, Darmstadt, Germany) dissolved in phosphate-buffered saline (PBS). The cells were incubated for a further 24 hours for recovery. Medium was removed, and cell viability was tested with MTT (3-(4,5-dimethylthiazol-2-yl)−2,5-diphenyltetrazolium bromide, Carl Roth GmbH, Karlsruhe, Germany). MTT (100 µL 0.5% (w/v)) dissolved in phosphate-buffered saline (PBS) was added to each well and the plate incubated for 3 h at 37 °C and 5% CO_2_. Cells were lysed by adding 100 µL isopropanol and the A_630_ subtracted from A_570_ automatically in the plate reader (TriStar2 LB942, Berthold Technologies, Bad Wildbad, Germany). Medium containing 6 mM cysteine did not cause any reduction of MTT.

### BY-2 Tobacco cells

BY-2 cells (kindly provided by Dr. C. Langenbach, Institut f. Biologie III, RWTH Aachen University) were grown in modified MS Medium (4.3 g L^−1^ MS basal salt mixture (Duchefa Biochemie, Haarlem, Netherlands), 30 g L^−1^ sucrose, 0.2 g L^−1^ KH_2_PO_4_, 0.2 mg L^−1^ 2,4-Dichlorophenoxyacetic acid, 1 mg L^−1^ Thiamin Hydrochloride, 100 mg L^−1^ Myo-Inositol, pH = 5.8 with KOH (all chemicals were purchased from Carl Roth, Karlsruhe, Germany)) shaken in the dark at 90 revolutions min^−1^ for 7 days. Cells were treated with thiosulfinates for 1 hour and then incubated for 15 min with 0. 5% Evans Blue (Sigma), unbound dye was removed by washing. Dye bound to dead cells was solubilized in 50% methanol with 1% SDS for 30 min at 50 °C and quantified by absorbance at 600 nm in a plate reader. Negative controls were not treated with thiosulfinates and positive controls were heated to 99 °C for 30 minutes^54^. Negative and positive controls were set as 0% and 100%, respectively. Data are presented as means with standard deviations of four replicates.

### *Arabidopsis* seedling root assay

*Arabidopsis* seedling root assay was performed after Borlinghaus *et al*.^3^ Surface-sterilized *Arabidopsis thaliana* seeds (Col-0, *pad2* and *gr1*) were sown on sterile filter papers and placed on Murashige & Skoog (MS) solid medium. The Petri plates were tilted to an angle of approx. 70° to ensure root growth according to root gravitropism. After three days of cultivation, filter papers were transferred to MS medium that contained different amounts of thiosulfinates. After 3 days treatment seedlings were photographed and the root length was measured.

## Conclusions and Perspective

Based on allicin (diallylthiosulfinate, DATS) as the lead compound, a comprehensive investigation of the antibiotic properties of molecules with a thiosulfinate functional group and symmetrical side chains modifying their physical properties (log *P*, diffusivity) showed that:

- All thiosulfinates tested exhibit a degree of antimicrobal activity comparable to allicin, but absolute activities depended on the conditions used in the individual tests.
- Except for DBTS all the thiosulfinates tested were more heat-stable than allicin.
- For allicin, activity by diffusion through the gas phase could be detected after as little as one hour of exposure. This may suggest a possible use for the treatment of lung diseases because conventional antibiotics are not volatile. Furthermore, uses in agriculture, e.g. fumigation of soils and treatment of seeds against seed-borne diseases can be considered.
- Antimycotic activity was higher than antibacterial activity for the thiosulfinates tested.
- The hypersusceptibility of the *Saccharomyces cerevisiae Δyap1*, *Δglr1*, *Δzwf1*, *Δgnd1*, and *Δtrx2* and the *Arabidopsis pad2* and *gr1* mutants suggests that, like allicin, the other thiosulfinates may attack the glutathione pool and glutathione metabolism in target cells.
- The synthesis method employed^32^ can be used for very many natural and non-natural disulfides to create the more reactive electrophilic sulfur centre of the thiosulfinate functional group and opens up the possibility to investigate very many novel compounds, with potentially improved properties compared to the lead substance allicin.

Although allicin and the analogue thiosulfinates are natural products, nearly all to be found to greater or lesser extent in the common foodstuff garlic, any strategies to develop thiosulfinate-containing formulations for use in medicine and agriculture must of course be accompanied by detailed economic feasibility analysis and toxicology testing, not least of all from the view of potential exposure of agricultural workers when plants are being treated on a field scale. Certainly, consuming garlic itself is generally considered to be health-promoting and the low levels of oxidative stress which result through consumption cause activation of phase 2 protection enzymes, such that garlic is considered physiologically to be an antioxidant foodstuff  ^2^. The results presented here contribute to knowledge of the effects of thiosulfinates on a wide range of organisms including procaryotes and eucaryotes and suggest that thiosulfinates, alone or possibly in combination with other substances, could be developed for specific applications in medicine or agriculture.

### Data availability statement

Data will be made available upon request.
